# Dynamic Gene Clusters Mediating Carbapenem-Resistant *Acinetobacter baumannii* Clinical Isolates

**DOI:** 10.3390/antibiotics11020168

**Published:** 2022-01-28

**Authors:** Samy Selim, Osama Ahmed Faried, Mohamed S. Almuhayawi, Osama A. Mohammed, Fayez M. Saleh, Mona Warrad

**Affiliations:** 1 Department of Clinical Laboratory Sciences, College of Applied Medical Sciences, Jouf University, Sakaka 72341, Saudi Arabia; 2 Medical Microbiology and Immunology Department, Faculty of Medicine, Beni-Suef University, Beni-Suef 62513, Egypt; sadomm2008@gmail.com; 3 Department of Medical Microbiology and Parasitology, Faculty of Medicine, King Abdulaziz University, Jeddah 21589, Saudi Arabia; msalmuhayawi@kau.edu.sa; 4 Department of Clinical Pharmacology, Faculty of Medicine, Ain Shams University, Cairo 11566, Egypt; osamaabbass@med.asu.edu.eg; 5 Department of Clinical Pharmacology, Faculty of Medicine, Bisha University, Bisha 61922, Saudi Arabia; 6 Department of Medical Microbiology, Faculty of Medicine, University of Tabuk, Tabuk 71491, Saudi Arabia; fsaleh@ut.edu.sa; 7 Department of Clinical Laboratory Sciences, College of Applied Medical Sciences at Al-Quriat, Jouf University, Al-Quriat 77454, Saudi Arabia; mfwarad@ju.edu.sa

**Keywords:** dynamic gene, repetitive extragenic palindromic, imipenem, carbapenem-resistant, *Acinetobacter baumannii*, nosocomial infection

## Abstract

*Acinetobacter baumanni* (*A. baumannii*), a nonfermenting Gram-negative bacterium, has recently been associated with a broad range of nosocomial infections. To gain more meaningful insight into the problem of nosocomial illnesses caused by the multidrug-resistant (MDR) *A. baumannii*, as well as the factors that increase the risk of catching these infections, this investigation included a total of 86 clinical *A. baumannii* infections. Repetitive extragenic palindromic (REP)-PCR was used to investigate imipenem-resistant *A. baumannii* isolates for dynamic gene clusters causing carbapenem resistance. Four distinct *A. baumannii* lineages were found in the REP-PCR-DNA fingerprints of all isolates, with 95% of the samples coming from two dominant lineages. Imipenem, amikacin, and ciprofloxacin were less effective against genotype (A) isolates because of enhanced antibiotic tolerance. Lastly, to gain more insight into the mode of action of imipenem, we explored the binding affinity of imipenem toward different *Acinetobacter baumannii* OXA beta-lactamase class enzymes.

## 1. Introduction

There has recently been a rise in the prevalence of *A. baumannii*-related skin and soft-tissue infections (e.g., wound infections, urinary tract infections, and secondary meningitis [1,2]. Despite this, ventilator-associated pneumonia and bloodstream infections are the leading causes of mortality among patients in intensive care units [3]. *A. baumannii* may enter the body via a variety of routes, including intravascular catheters, mechanical ventilators, and even open wounds. Prolonged hospitalization, male gender, and older age all play a role in *A. baumannii*-induced infections [4,5].

Additional reports have shown *A. baumannii* to cause community-acquired diseases, such as pneumonia and bacteremia. Skin, soft-tissue, and eye infections and endocarditis are also potential *A. baumannii* community-acquired diseases [6,7]. Pneumonia caused by community-acquired *A. baumannii* is much more dangerous than that acquired within the hospital; it is usually fulminant (resulting in death within 8 days of diagnosis), and fatality rates may reach 60% [8]. Acquiring pneumonia in the community occurs most often in tropical and subtropical areas during the summer months [8].

As a relatively new problem, *A. baumannii* has been linked to infections after injuries in combat zones, such as Iraq and Afghanistan, or following the Marmara earthquake in Turkey [9,10]. Anecdotal evidence suggests that morphine may increase the risk of *A. baumannii* infection after a battle or crush injuries as a result of its immunosuppressive effect [11]. Instead of being the result of a causal link, *A. baumannii* outbreaks may be a reflection of the great stress on hospital emergency rooms, leading to a failure in infection control measures and the subsequent epidemic spread of *A. baumannii* [12].

*Acinetobacter baumannii* infections may be deadly, with fatality rates ranging from 8% to 35%, depending on the strain and the infected host [8]. Different *A. baumannii* strains may have different levels of pathogenicity, as demonstrated by two studies using the mouse model of pneumonia [13]. An MDR strain isolated from the blood culture of a patient with nosocomial pneumonia showed 80% mortality in mice, whereas a strain causing meningitis showed only 13% mortality [14,15]. The expression of particular virulence factors and determinants may explain differences in mortality. In a mouse model of pneumonia, two strains with an identical PFGE profile—one mucoid from cerebrospinal fluid and the other nonmucoid from a ventriculoperitoneal catheter—had death rates of 48% and 19%, respectively [16].

The predominance of certain *A. baumannii* lineages has been related to the MDR phenotype of infecting strains [17]. However, it is not yet apparent whether epidemic strains acquired the MDR phenotype or if the MDR phenotype is necessary for individual strains to become pandemic [12]. Antimicrobial resistance in *A. baumannii* has steadily grown since the 1970s, when the great majority of strains were susceptible to frequently used antibiotics. Most isolates (depending on nation, hospital, medical department, and clinical sample) became MDR by 2007, including carbapenem resistance, which was long regarded as the cornerstone against MDR *A. baumannii* infections [18]. Carbapenem-resistant bacteria have been found in isolates from both military and civilian establishments [19,20].

Colistin seems to be the most effective antibiotic in vitro against MDR *A. baumannii*, although colistin usage has been linked with a variety of adverse effects and is not suited for treating all infections [8]. Some locations are seeing strains resistant to all known antibiotics resulting from the spread of colistin resistance around the globe [21,22]. At the same time, findings of carbapenem-resistant *Acinetobacter* spp. in cattle and other animals [23,24] and in the Seine River in Paris, France, suggest that resistance is spreading to the population [25]. In the last few decades, *A. baumannii* has developed a resistance to a wide range of antimicrobial drugs. The ability of this bacterium to acquire resistance genes, frequently through horizontal gene transfer, is a major factor in this ability [26]. *A. baumannii’s* effectiveness as a nosocomial pathogen may be linked to its ability to acquire the MDR phenotype, according to recent research [27].

In hospitals, bacterial infections are a leading cause of illness and death [28]. Bacteremia, pneumonia, urinary tract infection, and peritonitis are only some of the possible consequences that might arise from these illnesses [29]. Because of concerns about colistin’s toxicity and the emergence of newer antibiotics with better safety profiles during the last 30 years, its use has been restricted [30,31]. Colistin’s strong effectiveness against MDR *A. baumannii* has rekindled interest in its use, despite the dearth of novel antimicrobial drugs and the growing prevalence of this pathogen.

Beta-lactam antibiotics have been widely used for the treatment of *A. baumannii* infections. However, due to the widespread resistance to beta-lactams in *A. baumannii* infections, carbapenems have been currently considered as the most effective therapeutic agents for treatment of *Acinetobacter* infections. Carbapenems represent a subclass of antibiotics belonging to the class of beta-lactam antibiotics, and they have been recently considered for the treatment of severe multidrug-resistant bacterial infections [32,33,34,35]. Nevertheless, the production of carbapenemases, also known as beta-lactamases or oxacillinases (OXA), that hydrolyze carbapenem antibiotics, has been shown to be the major paradigm causing carbapenem resistance in *A. baumannii* infections, leading to a serious risk to public health [36,37,38,39]. Carbapenemases are categorized using the Ambler classification method into four classes A–D. Class A, B, and D carbapenemases (serine beta-lactamase) are recognized by the serine residue in their active sites, while class B carbapenemases (metallo beta-lactamase) are metallo-proteinases that require Zn for their activity [40,41,42,43]. In *Acinetobacter* spp., there are more than six OXA carbapenemases have been identified, namely, OXA-23, OXA-24/40, OXA-51, OXA-58, OXA-143, and OXA-235 [44,45]. The hydrolytic mechanism of OXA classes is still elusive, except for OXA-23 which has been previously reported for meropenem [45]. Identification of the hydrolytic mechanism of antibiotics plays a critical role for the discovery of drug-like compounds. Toward this, the molecular modeling strategy is considered a potential tool to help in understanding the binding of specific small molecules and proteins [46,47]. 

To this end, we were able to type and molecularly explore antibiotic resistance elements in *A. baumannii* isolates from Saudi Arabia in this study, to better recognize epidemiological features of *A. baumannii* in nosocomial infection and the connection between antimicrobial resistance and gene clusters. Furthermore, we have in silico investigated and analyzed the interaction of imipenem with various *A. baumannii* OXA carbapenemases.

## 2. Materials and Methods

This cross-sectional study was carried out during the period from December 2021 to January 2022. A total of 86 different *A. baumannii* isolates were obtained from patients attending the outpatient clinics in the Prince Mutaib Bin Abdulaziz Hospital in Saudi Arabia. Bacterial isolates were processed in the Department of Clinical Laboratory Sciences, College of Applied Medical Science, Jouf University, Saudi Arabia.

### 2.1. Strain Conservation and Antimicrobial Susceptibility Testing

The Vitek GNI system (bioMerieux, Marcy l’Etoile, France) was used to identify *A. baumannii* isolates. An inoculum consisting of 10^4^ cfu per spot was used to determine the MICs of multiple antibiotics for these isolates. The methods used were the agar dilution method with Müeller–Hinton agar and the Kirby–Bauer disc diffusion method [48,49,50]. It is recommended to use antibiotics such as imipenem (IPM), meropenem (MEM), ciprofloxacin (CIP), levofloxacin (LVX), piperacillin/tazobactam (TZP), ceftazidime (CAZ), cefotaxime (CTX), cefepime (FEP), doxycycline (DO), and amikacin (AMK). Patients gave their written consent to have their health information preserved in the hospital database.

### 2.2. PCR Amplification of blaVIM1 and blaVIM2 Genes

A simple boiling approach was used to acquire bacterial DNA for genotypic testing. The nucleotide sequences were used to build *VIM1* (F: 5′–AGTGGTGAGTATCCGACAG–3′, R: 5′–ATGAAAGTGCGTGGAGAC–3′) and *VIM2* (F: 5′–ATGTTCAAACTTTTGAGTAAG–3′, R: 5′–CTCAACGACTGAGCGATTG–3′) primers. The *bla_VIM1_* (261 bp) and *bla_VIM2_* (798 bp) genes were identified using optimized PCR. A 20^−l^ mixture of the primary solution was made with a PCR kit containing the following ingredients: 10× polymerase-cyclase buffer, 0.4 µL of dNTP, 1.25 µL of MgCl_2_, 0.2 µL of Taq DNA polymerase, and 13.55 mL of distilled water. The PCR procedure was run for 35 cycles for initial denaturation at 94 °C for 5 min before 35 cycles of 30 s at 94 °C, 40 s at 55 °C, and 50 s at 72 °C, with a final 5 min extension at 72 °C. The accuracy of the PCR products was checked using agarose gel electrophoresis in TBE buffer with a 1.5% concentration. Finally, the PCR products were visualized using UV light and gels stained with ethidium bromide [51].

### 2.3. Repetitive Element Sequence-Based PCR (REP-PCR) 

REP-PCR typing was used to identify the common REP types among *A. baumannii* isolates. The following primers were used for REP typing; F: 5′–IIIGCGCCGICATCAGGC–3′ and R: 5′–ACGTCTTATCAGGCCTAT–3′ [52]. The final volume of the reaction was adjusted to 25 µL. Reactions were carried out with the following components: 2.5 µL of 10× PCR buffer, 1.25 U of Taq DNA polymerase (Fermentas, UK), 0.8 µL of 2 mM mixed deoxynucleotide triphosphate, 1.5 µL of 25 mM magnesium chloride, and 5 µg of template DNA. The primers and template DNA were prepared as follows: 10 min of denaturation at 94 °C, 30 cycles of denaturation at 94 °C for 1 min, annealing at 45 °C for 1 min, and extension at 72 °C for 1 min; the amplification reaction was carried out by a thermal cycler (Bio-Rad, CA, USA. Followed by 16 min of final extension at 72 °C. On agarose 2% *w*/*v* gels, samples of each PCR end-product were examined.

### 2.4. Ethical Considerations

Approval was obtained from the Research Ethics Committee, Jouf University (Ethical Approval No. 3-04-43) and the Research Ethics Committee, Qurayyat Health Affairs (Registered with NCBE, Reg NO: H-13-S-071; Saudi Arabia; Project No. 111). The procedures used in this study adhere to the tenets of the Declaration of Helsinki. In this study, a written consent form was obtained from each patient.

### 2.5. In Silico Molecular Modeling Study

The binding affinity of imipenem to the OXA beta-lactamase binding site was explored by in silico molecular modeling using the Molecular Operating Environment software (MOE, 2015.10, Chemical Computing Group ULC, Tokyo, Japan. There are several crystal structures for the different OXA beta-lactamases available in the Protein Data Bank (PDB). To intensely explore the binding affinity of imipenem toward the OXA beta-lactamase protein, we extensively investigated the binding of imipenem toward the different available crystal structures of the OXA beta-lactamase protein. The 3D crystallographic structures of OXA proteins (PDB codes *3fyz, 4jf4, 4k0w, 4zdx, 5iy2, 6skq, 6skr,* and *7khq*) were obtained from the PDB website (http://www.rcsb.org/pdb, accessed on 19 December 2021). The unnecessary chains and water molecules were removed. The protonated 3D proteins were prepared for the docking studies using the protonate 3D protocol in MOE with default options. The 2D structure of imipenem was obtained using the Chemdraw program. For energy minimization and geometry optimization, the Conf Search module in MOE was applied; all the partial charges were automatically expressed, and the MMFF94x force field was used. Validation of the modeling protocol was achieved by redocking the original co-crystallized ligand to the binding site. For the validation step, the London dG scoring function and Triangle Matcher placement protocol were applied. The validated protocol was then used to dock imipenem in the OXA beta-lactamase binding site to assess its binding affinity. The obtained results were assessed, and the poses with high ligand–enzyme binding affinity were selected for energy assessment [53,54,55,56,57,58,59].

### 2.6. Data Analysis

The data were analyzed using the chi-square test, *Z*-test, and two-way ANOVA. The similarity matrix was calculated with the Pearson product-moment correlation algorithm. The unweighted pair group with mathematical average method was used for dendrogram construction.

## 3. Results and Discussion

**Figure 1:**
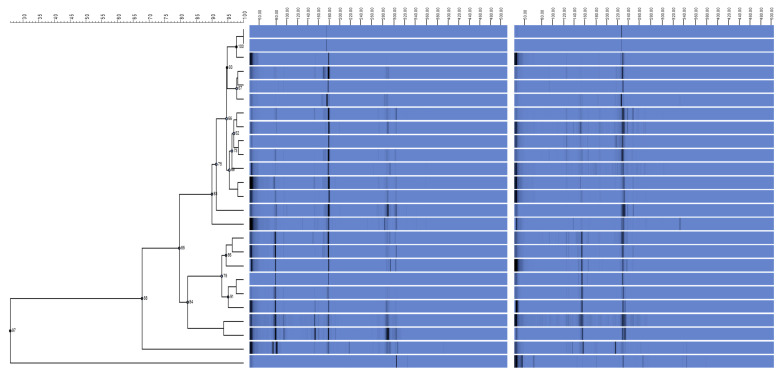
*Acinetobacter baumannii* relationships are depicted in this dendrogram. Using the unweighted pair group method of arithmetic averages of *Acinetobacter baumannii* REP-PCR DNA fingerprints, the dendrogram was built. Imipenem resistance profiles, *Acinetobacter isolates*, and REP-PCR fingerprints are all displayed. The dotted vertical line indicates the 90% similarity threshold.

Table 1 lists the antibiotic resistance profiles of 86 isolates of *A. baumannii*. Ciprofloxacin was the most effective antibiotic in the fight against *A. baumannii* (29.1%). Imipenem had the highest level of resistance among all isolates tested (89.5%). Using REP-PCR, it was discovered that imipenem-resistant *A. baumannii* isolates were distributed in five clusters, A, B, C, D, and E, with distribution rates of 45.5% (*n* = 35/77), 26% (*n* = 20/77), 19.5% (*n* = 15/77), 5.2% (*n* = 4/77), and 3.9% (*n* = 3/77), respectively. REP fingerprinting failed to discriminate between the REP patterns of 11 *A. baumannii* isolates (Figure 1).

Introducing antibiotic-resistant isolates from external sources, such as patients transferred from other institutions, could be a possible cause of antibiotic-resistant *A. baumannii* development [60]. REP-PCR DNA fingerprints of *A. baumannii* indicated two major lineages denoted genotypes (A) and (B) upon initial analysis. Antibiotic resistance may have evolved in the local genotype (D) isolates. It is possible that the bacteria evolved as a result of the increasing use of antibiotics, which exerted selection pressure [61]. Despite this, all genotype isolates had comparable REP-PCR DNA fingerprints and could have developed outside of a hospital setting. Antibiotype profile isolates are becoming increasingly common, which raises the possibility that they could evolve in situ into organisms with resistance qualities on par with genotype isolates [62]. The remarkably consistent REP-PCR fingerprints of Genotype 1 suggest the stability of the REP element positions in the bacterial genome and Genotype 4 isolates over the last decade. There were also consistent REP-PCR DNA fingerprints among distinct antibiotype profiles within a genotype, which shows that resistance acquisition may not have entailed gene insertion or deletion that affected the placement of the REP sequence elements. *Acinetobacter* spp. may have developed resistance to antibiotics because of other processes, such as gene mutation and gene silencing. REP-PCR amplification, on the other hand, may not have included any genomic area containing the acquired genes that contributed to antibiotic resistance [63].

### In Silico Molecular Modeling Study

Molecular docking is considered a valuable computational approach to evaluate the binding affinity of a drug toward a targeted protein [64]. To date, the exact hydrolytic mechanism of carbapenems by different OXA carbapenemases is still unexplored. To gain insights into the mechanistic mode of action of carbapenems toward OXA carbapenemases, we extensively investigated the binding affinity of imipenem toward the active site of different plausible models of OXA proteins [65,66,67,68,69,70,71]. 

We performed in silico molecular modeling studies for several 3D crystal structures which are available for OXA proteins in PDB, and the results were extracted for comparison (Table 2). The selection of the best poses was based on the ability of imipenem to possess the major interactions for binding to the protein. The applied docking protocol was initially validated through redocking of the co-crystallized ligand to the binding site of the different OXA proteins. This step was successfully carried out for OXA protein crystal structures within a 1.0–1.5 Å of the original co-crystallized ligand. In all presented cases, the selected poses demonstrated the main interactions occurring in the original ligand–protein interactions. 

**Figure 2:**
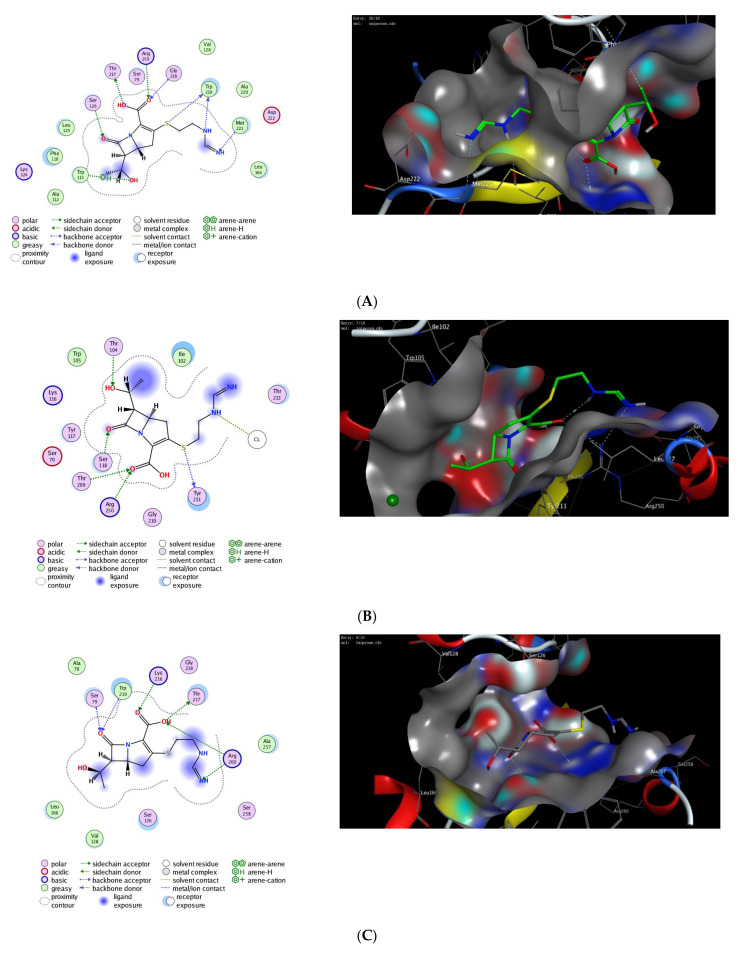

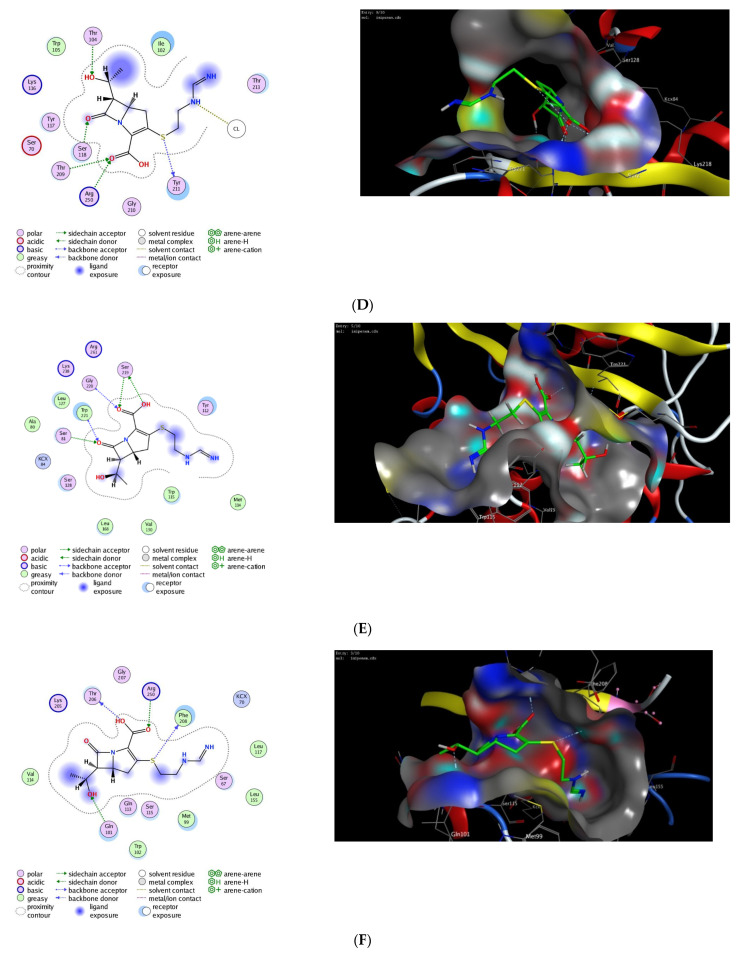

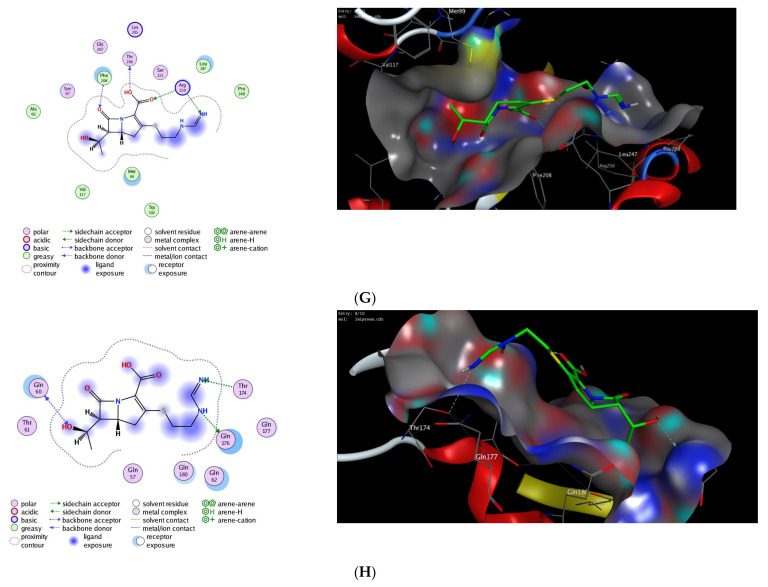
The 2D and 3D molecular docking interactions of imipenem (green in 3D interactions) with OXA proteins: OXA-23 (**A**), OXA-48 (**B**), OXA-23 A220 (**C**), OXA-24 (**D**), OXA-143 (**E**), OXA-655 (**F**), OXA-10 (**G**), and OXA-51 (**H**). The hydrogen bonds are illustrated as dotted blue arrows; (C atoms, green; S atoms, yellow; O atoms, red).

Next, we applied the validated protocol to explore and evaluate the affinity of imipenem to bind to the active site of OXA proteins. The scoring results and interactions of the docking process are presented in the Table 2. The binding sites of OXA enzymes have been identified in the cleft regions [72]. The surface of the enzymes is occupied by water molecules, and the active sites contain a serine residue (Ser80) together with other amino-acid residues (Lys83, Ser127, Thr217, Trp222, and Arg260) [73]. Our results indicated that imipenem has a high binding affinity toward the active site of OXA protein with high docking scores. Furthermore, imipenem has the ability to bind to the binding site of OXA proteins via a set of hydrophobic and hydrophilic interactions (Figure 2, Table 2). Under validated docking conditions, imipenem demonstrated the ability to form the main interactions of the original co-crystallized ligand with high to moderate binding scores (Table 2). In addition, imipenem exhibited the ability to form extra hydrogen bonding interactions with residues in the active site of OXA protein. For example, the most favorable pose of imipenem demonstrated an additional hydrogen bonding interaction with Trp219 residue (OXA-23, PDB, *4jf4*), with Tyr211 residue (OXA-48 and OXA-24, PDB, *7khq and 3fyz*), and with Arg260 residue (OXA-23 A220 PDB, *4k0w*) (Figure 2, Table 2). The interaction between imipenem and OXA protein was thermodynamically favorable as indicated by the negative values of binding scores. The analysis of docked poses revealed the role of the hydrophobic interactions in improving the binding affinity of imipenem. In fact, imipenem exhibited hydrophobic contacts between the hydrophobic residues of the protein cavity and the hydrocarbon moiety of the scaffold (Figure 2). According to our results, OXA-23 is the most potent OXA enzyme to be further studied. Furthermore, these results revealed the characteristics of the binding efficiency of carbapenems (imipenem) to the different OXA enzymes. The obtained noncovalent complexes imply proper interactions with active amino-acid residues which lead to stability and high binding energy [74]. Overall, our molecular modeling studies reveal that the effect of imipenem could be attributed to its ability to bind to the active site of OXA proteins. Further extensive studies are still required to fully explore the dynamic hydrolytic mechanism of OXA enzymes toward carbapenems, which would help in identifying novel targets against *A. baumannii* infections.

According to a recent study, nosocomial infection has been shown to have a significant impact on patient mortality and morbidity [75]. It was more common for infections to be bacterial in origin in the early days, with the most prevalent sources of infection being the circulatory system, abdomen, urinary tract, and drain [76]. A higher infection rate was shown to be associated with prolonged use of parenteral feeding and time spent in the intensive care unit (ICU) [77]. Effective treatment of infections requires early detection and treatment for patients to ensure the best outcome. In selecting a preventive regimen, the prevalent bacterial isolates found in the hospital must be considered. Taking into account our findings, future research into infection risk and effective prophylaxis is warranted.

MDR Gram-negative and Gram-positive *Bacilli* are frequently seen in nosocomial infections [78]. These infections are linked to allograft acute rejection, reoperation after transplant, and abdominal infection. A patient’s prognosis is much worse with MDR Gram-negative *Bacillus* and is related to bacterial infections, renal impairment, stomach infection, and extended endotracheal intubation. Growing numbers of MDR Gram-negative *Bacilli* complicate clinical treatment.

## 4. Conclusions

Our in vitro and in silico molecular modeling study found a wide range of genes coding for imipenem in our area. *A. baumannii* strains also included genes linked to imipenem, which highlights the need to prevent and control their spread.

## Figures and Tables

**Table 1:** Comparison of drug resistance patterns of 86 *Acinetobacter baumanni* isolates against antimicrobial agents.

Antimicrobial Agent	*Acinetobacter baumannii* Isolates (*n* = 86)		*p*-Value *
	Susceptible	Resistance	
Imipenem	9 (10%)	77 (89%)	<0.05
Meropenem	18 (21%)	68 (79%)	<0.05
Ciprofloxacin	25 (29%)	61 (71%)	<0.05
Levofloxacin	13 (15%)	73 (85%)	<0.05
Piperacillin + tazobactam	23 (27%)	63 (73%)	<0.05
Ceftazidime	19 (22%)	67 (78%)	<0.05
cefotaxime	19 (22%)	67 (78%)	<0.05
Cefepime	16 (19%)	70 (81%)	<0.05
Amikacin	13 (15%)	73 (85%)	<0.05
Doxycyclin	15 (17%)	71 (83%)	<0.05
Cloistin	13 (15%)	73 (85%)	<0.05

* The *p*-value represents the difference in antibiotic resistance rates.

**Table 2:** Interactions and scores of the docking process of imipenem in OXA beta-lactamase binding sites.

Protein	PDB	Docking Score (kcal/mol)	Interactive Residues	
			Hydrophilic Interactions	Hydrophobic Interactions
OXA-24	*3fyz*	−10.39	Ser219, Arg261, Trp221, Lys218	Met223, Val130, Leu168, Met114, Trp115
OXA-23	*4jf4*	−13.21	Thr217, Ser126, Arg259, Gly218, Trp219, Met221, Trp113	Phe110, Leu125, Val128, Ala220, Leu166, Ala112
OXA-23 A220	*4k0w*	−11.86	Ser79, Trp219, Lys216, Thr217, Arg260	Ala78, Ala257, Val128, Leu166
OXA-51	*4zdx*	−8.52	Gln60, Thr174, Gln176	--
OXA-143	*5iy2*	−10.29	Ser81, Trp221, Gly220, Ser219	Leu127, Ala80, Leu168, Val130, Trp115, Met114
OXA-655	*6skq*	−9.82	Gln101, Phe208, Arg250, Thr206	Val114, Trp102, Met99, Leu155, Leu117
OXA-10	*6skr*	−8.46	Thr206, Phe208, Arg250	Ala66, Leu247, Pro248, Val117, Met99, Trp102
OXA-48	*7khq*	−10.73	Thr104, Thr209, Ser118, Arg250, Tyr211	Trp105, Ile102, Val120, Leu247

## Data Availability

Not applicable.
